# Surgical Planning for the Treatment of a Patient with Multiple, Secondary, Intracranial Echinococcal Cysts

**DOI:** 10.1055/s-0037-1603321

**Published:** 2017-05-22

**Authors:** Ahmet Tuncay Turgut, Mehmet Turgut

**Affiliations:** 1 Department of Radiology, Hacettepe University Faculty of Medicine, Ankara, Turkey; 2 Department of Neurosurgery, Adnan Menderes University Faculty of Medicine, Aydın, Turkey

We read with interest the article by Chatzidakis et al entitled “Surgical planning for the treatment of a patient with multiple, secondary, intracranial echinococcal cysts” 1 and congratulate the authors on their original study. Today, hydatid disease is not a major public health problem in Europe, but it is still a serious disease in some regions of the world including Mediterranean countries such as Turkey and Greece. 2 3 4 5 In hydatid disease, multiorgan involvement is common, but the concomitant involvement of heart and brain is quite infrequent. 1 2 3 4 5 6 7 8 For the sake of completeness of the information presented, we would like to add the following important points to the discussion:

1. In the presented case, it is very crucial to know that multiple intracranial cysts were developed “secondary” to cardiac embolization through the vascular system, although the exact location of the cyst within the heart was not reported by the authors. In cases of cardiac hydatidosis, the most common location is the left ventricle, possibly due to dominance of the left coronary artery. 6 7 As a rule, the ruptured hydatid cyst in the left ventricle of the heart results in systemic emboli in organs supplied by the aortic circulation including brain tissue, while right ventricular cyst rupture leads to pulmonary embolus. 8
2. Cerebral hydatid cyst can be a single fertile “primary” focus due to migration of the larvae to the right heart through the inferior vena cava and the brain in case of a patent septal cardiac foramen or arteriovenous channels in the lung or multiple sterile metastatic lesions “secondary” due to spontaneous, surgical, or traumatic rupture of a primary cyst localized in the left compartments of the heart. 4 5 7 9 Thus, the presented patient is a typical case of multiple cerebral cysts secondary to rupture of a primary cyst of the heart. In our review, in 2014, we documented a total of 41 such cases as a complication of cardiac hydatidosis in the world literature. 5
3. The authors have not documented the surgical technique used in their case, but the best choice for removal of the cerebral cysts is the Dowling-Orlando technique. 2 3 4 5 Technically, however, complete removal of multiple cysts was usually difficult and radical extirpation of the cyst was not possible without rupture, although there is no risk of rupture in the infertile secondary cysts.
4. On computed tomography and magnetic resonance (MR) imaging, hydatid cyst has a density similar to that of cerebrospinal fluid, and there is no peripheral enhancement of the cyst. 9 Characteristic radiological findings of cerebral hydatidosis are well-defined, smooth thin-walled, spherical, homogeneous cystic lesions with no contrast enhancement, no calcification, and no surrounding edema. Importantly, it has also been suggested that the differentiation of “fertile” and “sterile” cysts may be possible with proton MR spectroscopy. 10
5. Based on the results of a meta-analysis of cases of cerebral stroke as a complication of cardiac hydatidosis, 5 we suggest a possible explanation for midline shift in the left occipital lobe presenting with generalized seizures in the current case: existence of the infarcted cerebral tissue surrounding the hydatid cyst, rather than expansion of cyst within the left occipital lobe 2 weeks later.
6. As reported by the authors, antihelminthics including albendazole are recommended in patients with multiple cysts, though the efficacy of medical treatment is questionable in hydatidosis. 2 3 5
7. Last but not the least, we think that a careful investigation for the possibility of cardiac cyst as a source of embolism should be undertaken in patients with multiple intracranial hydatid cysts.

As you can guess, we convey these comments not as a criticism of *The Surgery Journal* but, instead, as a constructive process for editorial review.
